# Behavioural Correlates of Lemur Scent-Marking in Wild Diademed Sifakas (*Propithecus diadema*) in the Maromizaha Forest (Madagascar)

**DOI:** 10.3390/ani13182848

**Published:** 2023-09-07

**Authors:** Longondraza Miaretsoa, Valeria Torti, Flavia Petroni, Daria Valente, Chiara De Gregorio, Jonah Ratsimbazafy, Monica Carosi, Cristina Giacoma, Marco Gamba

**Affiliations:** 1 Department of Life Sciences and Systems Biology, University of Torino, 10123 Torino, Italydaria.valente@unito.it (D.V.);; 2 Groupe d’Étude et de Recherche sur les Primates de Madagascar (GERP), Fort Duchesne, Antananarivo 101, Madagascar; 3 Department of Sciences, Roma Tre University, 00146 Rome, Italymonica.carosi@uniroma3.it (M.C.)

**Keywords:** olfactory communication, scent mixing, sex-specific pattern, scent odour, lemurs

## Abstract

**Simple Summary:**

Many animal species use body odours and secretions to communicate with conspecifics. In this study, we observed wild lemurs (diademed sifakas) to understand how and where they deposit scent marks, and whether rank and sex influence the scent-marking behaviour. We found that lemurs deposit their scent marks by rubbing different parts of their bodies, and that the deposition pattern varied according to both sex and social status of the individuals. In particular, we observed that dominant individuals often deposited glandular secretions along with urine when marking, and that the most common areas for marking were the anogenital and chest regions, with chest rubbing being more frequent in dominant males. Males also showed longer and more complex scent-marking sequences compared to females. Moreover, we found that lemurs preferred tree trunks and marked at a similar height, regardless of age or sex. Our results suggest that this species uses a mix of glandular secretions and excreta to increase the probability of signal detection by conspecifics.

**Abstract:**

Scent-marking through odours from excreta and glandular secretions is widespread in mammals. Among primates, diurnal group-living lemurs show different deployment modalities as part of their strategy to increase signal detection. We studied the diademed sifaka (*Propithecus diadema*) in the Maromizaha New Protected Area, Eastern Madagascar. We tested whether the scent-marking deposition occurred using a sequential rubbing of different body parts. We also tested if glands (i.e., deposition of glandular secretions) were more frequently rubbed than genital orifices (i.e., deposition of excreta) by comparing different kinds of rubbing behaviour. We then investigated if the depositor’s rank and sex affected the sequence of rubbing behaviour, the height at which the scent-marking happened, and the tree part targeted. We found that glandular secretions were often deposited with urine, especially in dominant individuals. The probability of anogenital and chest marking was highest, but chest rubbing most frequently occurred in dominant males. Markings were deposited at similar heights across age and sex, and tree trunks were the most used substrate. Males exhibited long and more complex scent-marking sequences than females. Our results indirectly support the idea that diademed sifakas deploy a sex-dimorphic mixture of glandular secretions and excreta to increase the probability of signal detection by conspecifics.

## 1. Introduction

Animals perform a variety of behaviours to transmit crucial information to conspecific receivers [1] via acoustic [2], visual [3,4] and/or olfactory communication [5]. The latter has several benefits over other forms of communication because scent signals can persist and remain active over time without requiring inter-individual physical proximity and direct interactions [6,7]. Scent-marking is the deposition of scent odour mainly targeting substrates within the home range [8]. To provide such chemical signals, several mammalian species use different scent odour sources [8,9,10,11]. For example, a number of species scent-mark with multiple glandular secretions [12,13,14,15] to convey permanent traits (e.g., species, genetic makeup, sex), as well as transient states (e.g., health, social, reproductive status) to conspecifics [16]. Previous studies reported that, while both sexes express scent gland secretions (e.g., anogenital, perianal scent glands), others are exclusive to males and suggested to be sexually-selected signals (e.g., throat/sternal, antebrachial and brachial [11,14,17,18]). Animals also use scent odours from excreta like urine and faeces [19,20,21,22,23], supporting the implication of the excretory products in scent-marking. Even though a vast repertoire of scent sources has been identified across taxa (e.g., carnivores [24,25], lemurs [9,26,27]), their contributions to scent-marking has rarely been investigated in wild populations.

Because scent-marking communicates the depositor’s identity/quality [28,29], the deployment of scent odours is always performed strategically [30,31]. Several species generate composite olfactory signals/cues by combining scent odours from different sources [9,32,33,34,35,36], displaying complex and conspicuous eye-catching behavioural sequences [37,38]. For example, the ‘pasted’ scent marks of brown hyenas (*Parahyaena brunnea*) comprise two anal gland secretions from sebaceous and apocrine tissues, respectively, deposited within the same sequence scent-marking event [39]. These patterns are commonly designated as scent mixing [40,41]. The latter was suggested to be indicative of additional information from each scent odour and, together, the mixture is hypothesized to provide a more precise indication of individual condition [41,42]. A recent study suggested that scent mixing increases signal longevity, especially when ephemeral odours are mixed with long-lasting (fixative) ones, thus, probably linked to a strategy for optimizing signal detection over time [43]. Other studies also found that scent odours from different sources are chemically diverse [44]. In that case, scent mixing may be a self-advertisement strategy to provide multiple messages being deployed at one time [45]. Scent mixing may be critical for glandular marker species living in challenging habitats, in which weather conditions (e.g., heavy rain, wind, humidity, etc.) continuously alter the integrity of the deposited chemical signal and deteriorate its longevity.

For several primate species, including lemurs, urine serves as the main matrix of scent-marking [31,46]. In nocturnal species, urine-marking is much more frequent than glandular scent-marking [11], and the scent deposition pattern is simpler (see [47] for review). Although quantitative data are still lacking, for some lemur species, urine can be deposited in sequence with scent-glandular secretions like anogenital scent marks [31,37,47]. On the other hand, some group-living and diurnal species rely instead on a suite of chemically diverse specialized glandular secretions, encoding a wide range of information about the signaller [29,48,49]. In male ring-tailed lemurs (*Lemur catta*), for example, ante-brachial secretions can be deposited either singularly (i.e., pure scent) or in sequence with brachial scent odours, forming a composite, long-lasting olfactory signal [41]. Green et al. also reported that male ring-tailed lemurs investigated and sniffed mixed scent odours significantly longer, indicating a higher interest in scent mixtures [43]. Besides evidence of scent mixing in captive and semi-captive lemur species [40], there is still a lack of studies investigating this aspect in wild populations.

Among lemurs, sifakas are scent-oriented; both sexes have an anogenital gland, although only males show a sex-specific sternal gland [27,50] more developed in dominant (*P. edwardsi* [51], *P. verreauxi* [18,52]) than in subordinates individuals. This leads to dimorphism in males’ chest colour, with stained dominant males and clean-chested subordinates [14,18]. Scent glands from genital and sternal glands have been shown to contain diverse microbial communities, and that of sternal glands clearly differed between stained and clean-chested males (*P. diadema* [53]). Notably, the sternal gland is testosterone-mediated [18], and chest marking occurs more frequently in dominant than subordinate individuals [54]. Sifaka species are also among those that retain the ancestral characteristic of marking with urine instead of relying primarily on glandular marking like other diurnal species. Previous studies suggest that this dual odour production system should shift the focus to the effects of sociality on chemical complexity, regardless of the urinary or glandular matrix [49].

The diademed sifaka (*Propithecus diadema*) is a diurnal lemur living in female-dominated groups, in which a single male is dominant over other males [55,56]. Although the species is known to perform complex scent-marking from both excreted and scent-glandular secretions [55], the scent-marking behaviour remains little investigated. Indeed, depositor sex, social rank and the species’ reproductive schedule may affect the scent-marking rate [55], but nothing is known about the delivery pattern or the factors influencing scent-marking deposition. In particular, it is unclear to what extent scent-glandular secretions contribute to scent-marking deposition in this potentially glandular marker species. Most studies support the idea that scent-marking could be related to intrasexual competition, especially in adult males [55]. Territorial defence is also commonly proposed, but its purpose may differ between the sexes. Male sifakas may defend a specific area of their home range, presumably to advertise their presence and keep away any challenger who might intend to integrate into the group [55]. Females, known to compete for access to food, also mark on the food substrate as a form of territorial and resource defence [46]. To be effective and reach the target receptor, the signal deposition should be appropriate to ensure its detection and longevity. This Critically Endangered lemur [57] inhabits rainy and mountainous forested habitats, and despite possessing a rich vocal repertoire [58] relies on scent-marking for both territorial defence and for intrasexual competition [55]. Therefore, *P. diadema* can be an optimal candidate species to investigate the pattern of intraspecific scent-marking deposition.

This study explores the deposition pattern of scent-marking in a wild population of diademed sifaka. Based on the hypothesis that scent-marking is primarily involved in intersexual competition in male sifakas, as well as in mate attraction and resource defence in females, we expect to find a highly complex behavioural pattern that promotes signal longevity and detection, as well as sexual dimorphism in scent deposition. First, as *P. diadema* exhibits scent mixing, we expect a higher rate of scent marks deposited sequentially rather than in singular bouts. If this prediction is supported, we also expect urine marking to be highly deposited in association with scent gland secretions. Findings in this sense would support the hypothesis of the increased longevity of mixed odour signals. Second, as scent-marking occurrence is affected by the depositor’s social rank in most sifaka species [52,55], we also predict that the dominant depositors would show the highest individual rate for the frequently performed ‘scent-marking bouts’. Third, assuming that glandular marker lemurs heavily rely on scent-glandular secretions, we then predict that anogenital and chest rubbing will occur more frequently in adult males and that only the latter should be affected by males’ social rank, as found in other sifaka species (see *P. verreauxi* [52]). Results in this sense would suggest a role of sexual selection in shaping those signals. Fourth, because *P. diadema* occupies the lower part of the forest and mainly uses tree trunks when moving within the habitat [59], we expect tree trunks to be targeted by scent-marking at a higher rate than branches. Fifth, because scent-marking is an intraspecific communication channel, the scent-marking deposition should be performed at similar heights across individuals to intercept conspecifics’ attention effectively. Finally, because males have additional chest glands, they should display longer and more complex scent-marking sequences than females.

## 2. Materials and Methods

### 2.1. Study Site, Study Group and Data Collection

We studied diademed sifakas in the Maromizaha New Protected Area, a primary rainforest located in Central-Eastern Madagascar (18°56′49″ S–48°27′33″ E). Maromizaha is a mid-altitude rainforest, ranging from 800 to 1200 m above sea level, with an endemism rate of up to 77% [60]. We studied five habituated neighbouring groups of *P. diadema*, collecting scent-marking data from 35 individuals (Table 1) from April to November 2018 and from May to December 2019. We focused on a particular group for 3–4 consecutive days per week and then moved on to another group. During a typical day in the field, we reached a study group at its sleeping site between 06:00 and 07:30 h, before the sifakas started being active, and collected data on all group members until dusk, between 16:00 and 17:30 h [55]. We individually identified sifakas using natural marks (i.e., scars and fur colour pattern [55]) when lemurs were mainly patrolling the lower part of the forest and thus visible. Two researchers and two research guides observed the focal animals simultaneously to ensure the correct identification and obtain consensus about the observed behaviours and estimates [55]. Every observer followed a focal individual per day, annotating each behavioural sequence characterizing scent-marking, while observing each scent deposition. We estimated the height (in metres) at which lemurs deposited scent-marking and the substrate they targeted (trunk or branch). This methodology allowed us to continuously follow the focal animals during the entire observation session. Overall, we obtained a total of 870.5 observation hours using the all-occurrence sampling method [61]. We included in the analyses only those individuals sampled for at least seven days per season (at least 14 days in total). We also transformed data into rates by dividing the number of scent marks by the individual observation time to ensure the comparability of animals with different observation times.

As sex and dominance status are critical elements for interpreting variability in scent-marking deposition [55], we also recorded all aggressive behaviours during feeding and non-feeding interactions and used those data to assess the dominance hierarchy between individuals [55,56]. We constructed the dominance hierarchy between individuals by performing a steepness analysis [62] using the aggressive interaction dataset to assign the social rank among the group members. To calculate the David’s Score for each lemur (i), we summed the proportion of individual wins during interactions with another individual divided by the number of interactions with the other individual (w) and “w2”, the summed “w” values of those individuals with which “i” interacted. We also subtracted individual loss (l), representing the sum of that particular individual’s losses against the other individual, and l2, which represents the summed “l” values of those individuals with whom “i” interacted [55]. Because our target species show female dominance [56], we performed the analysis separately for males and females (as described in previous work [55]). Individuals with the highest David’s scores were labeled as Dominants, and those with lower David’s scores were labeled as Subordinates (Table 1).

Following previous work, we considered three age classes [55]. We classified those sifakas born during the data collection period as young. Sifakas born less than two years prior to data collection were considered sub-adults. Finally, we considered adults the remaining individuals, aged 3.5 years old and above at the time of the study.

### 2.2. Behavioural Definition and Data Preparation

In this study, we defined a ‘scent-marking event’ as the deposition of scents, through excretory products and/or various exudates, deployed simultaneously without interruption. We defined each behavioural component as a ‘scent-marking act’ or ‘sequence’ that may include an olfactory (scent) or non-olfactory (non-scent) element [63]. We considered olfactory elements to be scent marks left in small amounts and accompanied by body movements such as body part rubbing [8] or tree adhesion (e.g., urine marking [31]) Specifically, we considered urination and defecation as olfactory elements if depositors adhered the excretory product over the substrate and if they were performed in sequence with scent odours/secretions from deputed glands. We examined two forms of scent-marking [18,55]: scent-marking events targeting ‘clean’ substrates where we did not observe other scent marks during that sampling day (hereafter ‘marking’), and scent marks that at least partially overlapped a previously deposited scent mark (hereafter ‘overmarking’).

**Figure 1:**
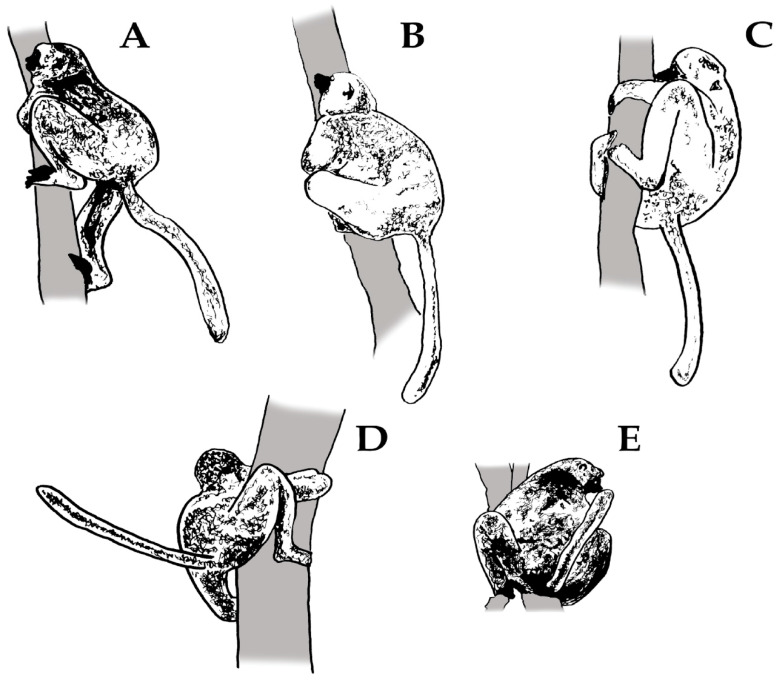
Patterns of singular and sequential bouts characterizing the deposition of scent-marks in diademed sifakas. Behavioural components of the scent-marking events are represented as (**A**) sniffing, (**B**) tooth marking, (**C**) chest marking/rubbing, (**D**) anogenital marking/rubbing and (**E**) urine and/or faeces marking.

We considered different categories of scent-marking bouts based on the olfactory elements deposited during a given scent-marking event (i.e., a mixture vs. pure scents). We labelled as ‘singular bouts’ those observations in which individuals deposited single olfactory elements (e.g., anogenital marking [GM]). However, we labelled ‘sequential bouts’ scent-marking acts or sequences (see Figure 1) containing at least two different olfactory elements (e.g., urine–anogenital marking [MU-GM]). Sequences were labelled according to the nature of the deposited scents: if scent-glandular secretions (SG) were deposited in sequence with urine marking, we labelled them as ‘SG-U’ (e.g., CM [chest marking]-MU-GM). We assigned them as ‘SG-F’ if scent-glandular secretions were deposited with faeces marking (e.g., CM-MF-GM). There were very few cases where urine and faeces occurred together within the scent-marking event (3.79%; *N* = 107 out of 2818 events).

### 2.3. Statistical Analysis

We ran all statistical analyses using R studio [64] and Behatrix software [65]. We investigated whether the species exhibited specific patterns when scent-marking, by comparing the individual rate (per minute) of different scent-marking bouts. To do so, we extracted the individual count of the sequentially and singularly deposited bouts (sequentially vs. singularly). Then, we calculated the individual rate by dividing the count by the individual observation time (minutes). For the sequentially deposited scent-marking bouts, we also explored if scent gland secretions were deposited with urine (SG-U) or faeces (SG-F). Finally, we calculated the individual occurrence rate (per minute) of each form of scent-marking bout by dividing the daily count by the total individual observation time.

We also investigated the frequency of occurrence of each ‘scent-marking act’. We created a separate dataset containing the behavioural sequence of the individual scent-marking events. We considered as a complete behavioural string all scent-marking events either containing a single or several ‘scent-marking acts’, namely tree-gouging (TM), chest marking (CM), and urine (MU), faeces (MF) and anogenital marking (GM). While analysing marking sequences, we pooled together all sequences shown by the individuals in our sample that belonged to a particular sex or age class and imported them into Behatrix software [65]. We used the behavioural strings statistics option in Behatrix to calculate the individual average frequency occurrence of each behavioural sequence, which represents the mean probability of each ‘scent-marking act’ to occur.

Next, we built ten generalised linear mixed-effects models (GLMMs). We ran four models for types of scent-marking bouts, four models for the frequency of each ‘scent-marking act’, one model for the scent-marking height and a last one to assess whether a scent-marking event was consistently deposited on either branch or tree trunk. We ran all models using the glmmTMB package, a flexible package that can handle zero-inflated data fitting a beta distribution [66].

We first tested the scent-marking rate as a response variable for our models. We set two initial models for males (GLMM1) and females (GLMM2) to assess if the scent-marking deposition was performed sequentially or singularly and whether deposition was affected by individual social rank. First, we used the interaction between the depositor social rank (Rank: DOM and SUB) and scent-marking bout (bout type: sequentially vs. singularly) as fixed factors. Second, to assess whether scent gland secretions were frequently deposited with urine or faeces, we built two other models for males (GLMM3) and females (GLMM4) in which we used the interaction between the social rank and the scent-marking bout (bout type: SG-U and SG-F) as fixed factors.

To investigate the delivery pattern of scent-marking, we also created another dataset containing the individual behavioural components of each. We then built models to assess if the frequency of occurrence of the shared scent-marking acts like urine (MU), faeces (MF) and anogenital marking (GM) and the males’ sex-specific tooth (TM) and chest marking (CM) significantly differed. We then investigated if the frequency of the males’ scent-glandular secretions (GM and CM) and females’ glandular marking act (GM) were affected by individual social rank. We set two models for males (GLMM5) and females (GLMM6), in which we used the mean frequency occurrence of marking acts as the response variable. For the two first models, we set the sequence of scent-marking (marking act) as a fixed factor. For the other two models (males: GLMM7 and females: GLMM8), we used the interaction between social rank and the scent-marking sequences (marking act: in males, GM, and CM; in females, GM and MU) as fixed factors. Therefore, we did not include TM, MU and MF scent-marking acts in the males’ interaction model to avoid multi-collinearity between the variables (only for the interaction model). For the same reason, we excluded MF in the female interaction model.

We also built a model (GLMM9) to verify if scent-marks were preferentially deposited on the trunk or the branch. We use the scent-marking rate (individual count of scent-marking divided by the total observation time) as the response variable. The substrate part targeted by scent-marking deposition (trunk vs. branch) was set as a fixed factor.

Finally, we used the last model (GLMM10) to assess if scent-marks were deposited across individuals at a similar height. To check individual variability, we use scent-marking height as the response variable (height) and the interaction between sex and rank as fixed factors.

The depositor identity and group membership were set as random factors for each of the ten models. We then developed a full (including the response variable and fixed and random factors) and a null model (including the response variable and the random factors). First, we verified if the residuals were homogeneous and normally distributed and checked the distribution plotted against the fitted values (“DHARMa” R package [67]). Next, we used the “performance” R package [68] to verify collinearity among predictors by extracting the variance inflation factors (VIFs), setting an inclusion threshold of VIF < 5 [69,70]. Then, we compared the significance of the full against the null model [71] using a likelihood ratio test (ANOVA with argument test Chisq [72]). Finally, we calculated the *p*-values for each predictor based on likelihood ratio tests between the full and the null model using the R function ‘drop1’ [73]. To assess the interaction between variables, we performed a pairwise Tukey test using the “multcomp” package in R [74].

To investigate the delivery pattern of scent-marking, we also created another dataset containing the individual behavioural component of each scent-marking bout (GM, CM, MU, MF and TM) and the investigatory sniffing behaviour (Table 2, Figure 1), considering marking and overmarking separately. We first pooled the data according to depositors’ sex, age, class and social rank and then imported the data into Behatrix. Next, we built a ‘transition matrix’, which refers to the ‘transition probabilities’ between the sequences of scent-marking behaviours. This method considers each behaviour sequence as a ‘state’; the probability of moving from one state to another is called transition [38]. To understand if the transition matrices between the components of the scent-marking event, which identify the relationships between the behaviours, are statistically significant (*p* < 0.05), we ran a randomization test using 10,000 random combinations. We then produced a transition diagram output across individuals (sex, social and age class) to determine the scent-marking delivery pattern (sequence of behavioural components). We set a cut-off threshold of 2% to exclude those infrequent transitions between behaviours.

## 3. Results

### 3.1. Occurrence Pattern of the Scent-Marking Bouts

Overall, we recorded 2818 scent-marking events, 68.70% (*N* = 1936) of which were deposited by males and 31.30% (*N =* 882) by females. Of the scent-marking bouts, 23.17% (*N =* 653) included a single marking event. Scent-marking bouts deposited sequentially and with at least two scent-marking acts accounted for 76.83% (*N* = 2165) of the scent-marking events. Also, most scent-marking bouts comprised a combination of excretory products and scent odours from gland secretions deposited in sequence (*N* = 1773; 62.92%). Another part of scent-marking deposition was from scent-glandular secretions without association with excreta, either deposited alone or in sequence (*N* = 1036; 36.76%). In a few cases (*N* = 9, 0.32%), the scent deposition involved odours from excretory products, urine or faeces without a combination with scent gland secretions.

**Figure 2:**
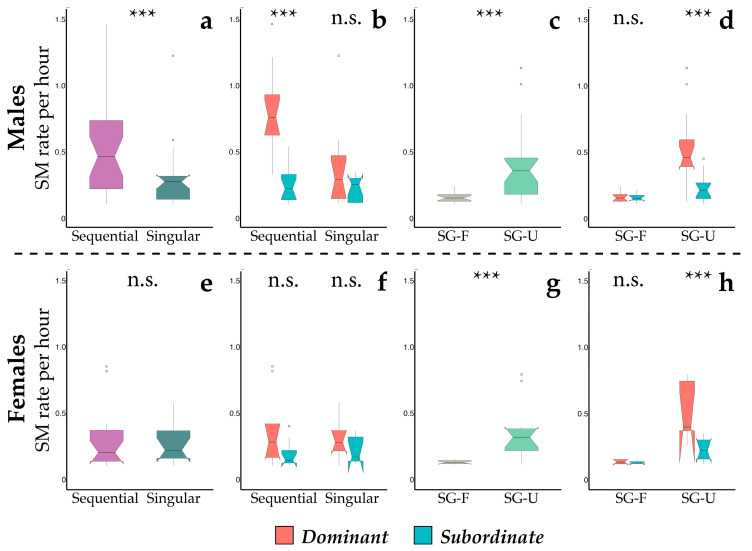
Boxplots showing the occurrence rate (per hour) of the scent-marking (SM) bouts in adult males (**a**–**d**) and females (**e**–**h**). We present here the patterns of singular (dark green) and sequential (purple) bouts in male (**a**,**b**) and females (**e**,**f**) and that of scent gland secretions (sequential bout) deposited in sequence with urine (light green) and faeces (grey) both in male (**c**,**d**) and female (**g**,**h**) depositors. Data of dominant (red) and subordinate (turquoise-green) individuals are pooled in (**a**,**c** for males; **e**,**g** for females). Three stars indicate statistical results with *p*-value < 0.001, and n.s. designates non-significant results with *p*-values > 0.05. Whiskers indicate 5th/95th percentiles, the horizontal line is the median, the box delineates the 25th/75th percentiles, and the open circles indicate outliers.

When we looked at the individual rate of scent-marking bouts, the full model significantly differed from the null one (GLMM1: χ^2^ = 41.525, df = 3, *p* < 0.001, Table 3) for males. Sifakas performed sequential bouts at a relatively higher rate, and they occurred more frequently in dominant than in subordinate males (Figure 2b). We did not find the same pattern for singular bouts (Figure 2a). Contrary to males, there were no significant differences between the singular and sequential bouts in females (Figure 2e), with the individual rate not affected by depositors’ social rank (Figure 2f). When considering scent gland secretions deposited in sequence with excretory products, the model found significant differences between scent-marking bouts deposited with urine (SG-U) and those with faeces (SG-F), both in male (GLMM3; full vs. null model: χ^2^ = 35.939, df = 3, *p* <0.001) or in female depositors (GLMM4; full vs. null model: χ^2^ = 23.882, df = 3, *p* < 0.001). Specifically, SG-U occurred at a significantly higher rate than SG-F (Figure 2c for males; Figure 2g for females). Also, SG-U bouts were highly affected by the depositor’s social rank, being highest in dominant males (Figure 2d) and females (Figure 2h) when compared to subordinate depositors.

### 3.2. Pattern of the Scent-Marking Acts’ Occurrence Frequency

**Figure 3:**
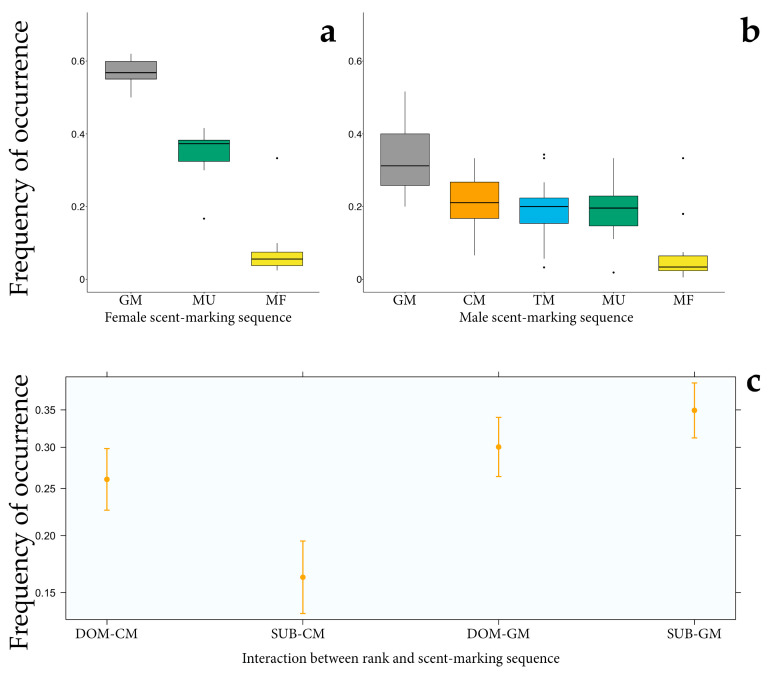
Plots highlighting the patterns of the occurrence frequency/probability of each sequence of scent-marking behaviour (MU: urine marking, MF: faeces marking, TM: tooth marking, GM: anogenital marking, CM: chest/sternal marking) within females’ (**a**) and males’ (**b**) scent-marking events and the effect of social rank on males’ glandular scent-marking act occurrence frequency (sternal and anogenital marking) (**c**). Colors denote particular scent-marking behaviors shown during the marking sequence. Whiskers indicate 5th/95th percentiles, the horizontal line is the median, the box delineates the 25th/75th percentiles, and the black dots indicate outliers. DOM: dominant depositor, SUB: subordinate individuals.

We observed significant differences in the occurrence frequency of each behavioural sequence within the scent-marking event either in male (GLMM5; full vs. null model: χ^2^ = 139.049, df = 4, *p* < 0.001, Table 4) or in female depositors (GLMM6; full vs. null model: χ^2^ = 75.170, df = 2, *p* < 0.001). Anogenital marking was the most frequently performed sequence in males (Figure 3b), followed by chest rubbing, urine marking, tooth marking and faecal marking. In females, anogenital rubbing also showed a higher probability of occurrence (Figure 3a), followed by urine marking and faeces deposition.

We also found that chest rubbing (CM)—as a male sex-specific sequence—was affected by male depositors’ social rank (GLMM7; full vs. null model: χ^2^ = 42.384, df = 3, *p* < 0.001) in which the occurrence frequency was higher in dominant than subordinate males (Figure 3c). In contrast to chest marking, the shared anogenital sequence occurrence did not differ between dominant and subordinate individuals. Similarly, in females the occurrence frequency of the anogenital marking act was not affected by the social rank of depositors.

### 3.3. Scent-Marking Height and Targeted Substrates

The model showed that scent-marking was deposited at a similar height amongst individuals (GLMM9; full vs. null model: χ^2^ = 2.820, *df* = 3, *p* = 0.420; mean height: 6 m). Neither the depositor sex (Tukey test; males vs. females: Estimate = −0.068; SE = 0.201; Z-value = −0.335; *p* = 0.737) nor the social rank (subordinates vs. dominants: Estimate = −0.293; SE = 0.221; Z-value = −1.325; *p* = 0.185) affected the height of scent-marking deposition. Regarding the targeted substrate part, scent marks were deposited more frequently on tree trunks than branches (trunk vs. branch: Estimate = 0.793, SE = 0.114, Z-value = 6.991, *p* < 0.001) (GLMM10; χ^2^ = 24.306, *df* = 1; *p* < 0.001).

### 3.4. Delivery Pattern of Scent-Marking

**Figure 4:**
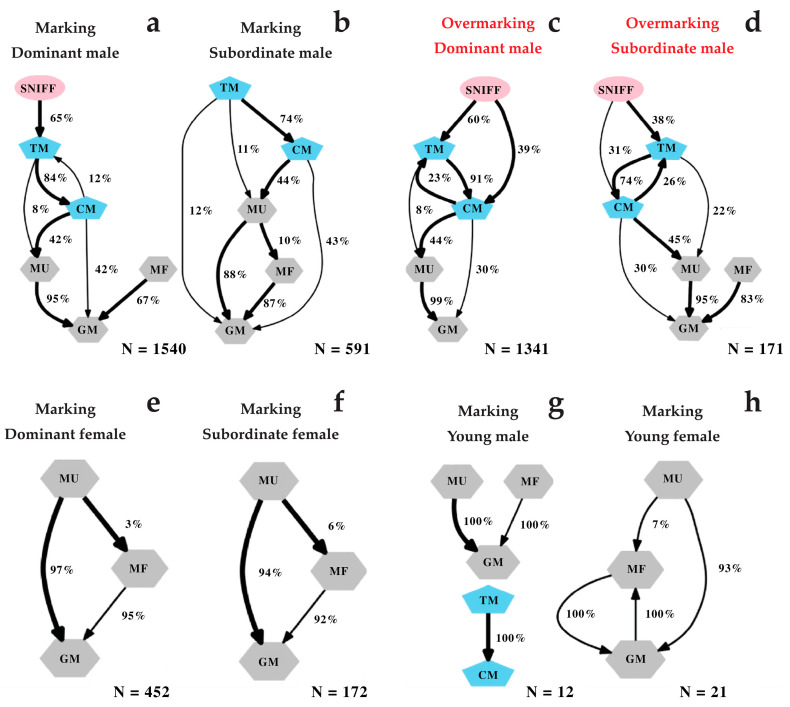
Flow diagrams showing the probability of succession between each behavioural sequence within a scent-marking event. Reported are the transition patterns of dominant (**a**) and subordinate males’ marking (**b**) and overmarking (respectively (**c**) and (**d**)), that of dominant (**e**) and subordinate females’ marking (**f**), as well as young males’ (**g**) and females’ (**h**) marking deposition. The coloured geometric shapes highlight a different group of behaviours: shared olfactory elements/sequences are represented by grey hexagons (MU: urine, MF: faeces, GM: anogenital markings); sex-specific sequences (both olfactory and non-olfactory elements) are highlighted by blue pentagons (CM: chest marking and TM: Tooth marking); the investigatory sniffing behaviour (non-olfactory element) is highlighted in pink ellipses (SNIFF). The numbers (in percent) beside each transition represent the probability of transitions between behaviours. The thick black arrows highlight statistically significant transitions (*p* < 0.05), and thin black arrows indicate non-significant transitions (*p* > 0.05); OVM: overmarking, M: marking; N: number of total transitions included.

The process by which sifakas deposited scent marks exhibited a complex configuration (Figure 4). In adult and high-ranking depositors, the majority of transitions (>66%) were statistically significant (Figure 4). We also found that males performed the longest and the most complex sequences (Figure 4a), which can contain up to four different scent-marking events: anogenital rubbing (GM), chest rubbing (CM), urine marking (MU) and faeces deposition (MF). These transitions can reach up to five sequences when including non-feeding tree gouging/tooth marking (TM). In dominant males, scent-marking always started by sniffing behaviour (sniffing the substrates bark). Deposition of drops of urine and anogenital rubbing could close the sequence. Females used mainly three sequences of olfactory elements, namely anogenital rubbing (GM), urine (MU) and faeces (MF) marking (Figure 4e). They exhibited a shorter and simpler behavioural diagram (Figure 4e: mostly started by urine marking and ended up with anogenital rubbing).

## 4. Discussion

We investigated the behavioural patterns of scent-marking deposition in a wild population of *Propithecus diadema* to understand the occurrence rate of different scent-marking events and the occurrence frequency of each scent-marking act, the height at which this behaviour is performed and the primarily targeted substrate part. We partially confirmed our first prediction that males of the species preferentially deposited a mixture of scent odours rather than pure scents. Specifically, males performed more sequences of different scent-marking behaviours than singular marking bouts, while this was not found for females. Our result is consistent with previous findings reporting scent mixing in another group-living lemur [41,43]. In male ring-tailed lemur (*Lemur catta*), odours from ante-brachial and brachial secretions were sequentially deposited to provide a composite chemical signal/cue to conspecifics [7,43]. In contrast to our first prediction, there was no difference in the rate of females’ sequential and singular bouts, which can be linked to the limited number of scent odours involved in their scent-marking deposition, as they mainly rely on anogenital and urine marking but rarely on faeces. However, males with up to four sources of scent odour (urination, anogenital and sternal glands, and rarely faeces) can consistently mix these odours to create a composite of chemical signals.

Our findings are also consistent with other studies showing the deployment of scent gland secretions in sequence with urine but rarely with faeces (*Propithecus verreauxi* [26], *Propithecus coquereli* [41], *Lemur catta* [31]). Also, in *P. diadema*, the deposition of scent gland secretions was frequently performed in close succession with urine, supporting our first prediction. This finding also supports the idea that, despite relying on scent gland secretions, sifaka species have maintained the ancestral urine marking behaviour typical in nocturnal lemurs [49]. The previous finding concluded that such a pattern of maintaining both glandular and urine marking could be linked to a convergent evolution in lemur chemical communication [49].

The reason why animals use scent mixing remains an open question. Literature suggests that scent mixing can be linked to a strategy to increase signal longevity [43,75]. A study using behavioural bioassays found that lemurs sniffed at mixed scents relatively longer than pure fresh scent signals and licked longer on mixed than on pure decayed scent, supporting a higher interest in the mixture of scent signals [43]. Other researchers suggested that combining scents and odours from different sources can increase the relative abundance of chemical component signals, potentially increasing signal detection and longevity [44,75]. In *P. diadema*, a study based on the glandular microbiomes found a clear difference between communities of genital and sternal gland secretions. The latter distinguished between stained/dominant and unstained/subordinate males [53]. Indeed, different microbiome communities across body parts may also imply chemical compound differences. In that case, mixing various odours with different chemical compounds may be essential to provide honest information about depositor identity [41,42] and attributes such as sex, fighting ability, and social rank or to communicate multiple messages [45] to conspecifics. Although our study was limited to behavioural observations, it suggests the importance of a mixture of odours from sternal and anogenital scent glands, and urinary scent in the species’ olfactory communication.

In support of our second prediction, sequentially deposited scent-marking and urine-deposited scent-gland secretions were highly affected by the depositor’s social rank, with higher rates shown by dominant individuals. The effect of social rank on scent-marking rate was also reported in previous studies on other lemur species (*P. edwardsi* [76], *P. verreauxi* [18,54], *Lemur catta* [77]).

Previous research showed that high-ranked male sifakas scent-marked more than subordinates [14,51,54,55,76]. In our study, sifakas showed marking sequences influenced by social rank more often. Depositors’ social status affected scent-marking dimorphically, especially in the rate of sequential bouts and urine-deposited scents, which may be related to a functional difference in scent-marking [51,54,55]. Male sifakas use scent-marking for territorial defence [55] and for monopolizing mating success [54], thus playing a role in intra- and inter-group communication. Previous studies suggested that dominant male sifakas often mate successfully with dominant females [46]. This mating pattern may explain the higher rate of frequently deposited odour mixtures in dominant males. Unlike males, females present themselves as a mating resource to be defended; therefore, they do not need to invest in scent-marking for this purpose. There is also increasing evidence that female lemurs compete for food, which is critical for their reproductive success. For example, females of *Lemur catta* were found to urine-mark their food resources to defend them from other groups [46]. This evidence could explain differences in the marking rate in dominant and subordinate females.

We also found that the deployment of scent odours from scent-glandular secretions occurred at a relatively higher frequency in adults than in subadults, supporting our third prediction. Anogenital rubbing (GM) was significantly higher in the adults of both sexes. Chest rubbing (CM) was the second most performed scent-marking act for males. Overall, *P. diadema* seems to primarily rely on scent-gland secretions, mostly associated with urine marking, to provide a mixture of scent odours to conspecifics. Again, this may allow conspecific receivers to gain a precise record of depositors’ characteristics, such as competitive ability and social and reproductive status [78,79]. In support of our third prediction, the males’ sternal marking act (CM) frequency was higher in dominant depositors, a relatively common scenario in sifaka species. In *Propithecus verreauxi*, sternal marking was performed more frequently by dominant (stained chest) than subordinate (unstained chest) individuals [52]. Moreover, dominant males are highly implicated in reproduction [54]. A higher occurrence frequency of the sex-specific marking act in dominant *P. diadema* may thus imply a selected function related to reproduction [14,52], probably linked to intrasexual competition between males [54,55] or female attraction. This explanation is congruent with evidence that the scent activity of the sternal gland is testosterone-mediated, as found in other sifaka species [14].

Like other sifakas, *Propithecus diadema* is a vertical clinger and leaper [59] that jumps between trunks when moving within its habitat, exploiting the lower strata of the forest [59]. Interestingly, scent-marking deposition was performed mainly at the lower part of the forest (mean = 6 m from the ground) and at a similar height across individuals, especially targeting substrate trunks and rarely branches. In this study, the lack of individual variability in the height at which scent marks were deposited and the fact that scent-marking targeted a highly used substrate part (trunk) suggest a strategy to intercept conspecifics’ attention to transmit information through mixed scent signals. A recent study also reported that our target species patrolled its home range throughout the day, and scent-marks were deposited non-randomly, targeting the contested areas of the home range, including the peripheral and the overlapping zones between neighbouring groups, in a way to increase signal detection by conspecifics [55]. This result supports the hypothesis that lemur scent-marking is an intraspecific communication system [18,51,76,77,80].

Also, the number of scent odour sources involved in scent deposition is a determinant factor for dictating the pattern of scent-marking [38]. We found that males with additional sex-specific sternal glands exhibited longer transition diagrams than females, displaying one to four sequences (faeces, urine, sternal and anogenital marking). The sequence can reach five behavioural components if we consider the non-feeding tree gouging. In contrast to males, females rely on the shared anogenital gland and urine marking (rarely faeces) to scent mark and display a simpler transition with one to three sequences. One exciting pattern of *P. diadema* scent-marking is that all possible scent-marking components can be deposited simultaneously, especially in males, where an individual may repeat some of the behaviours within a single scent-marking event (especially TM, CM and even GM). This is always the case when the scent deposition is related to overmarking.

Most studies on sifaka species also found evidence that males frequently overmark female deposits [52,55]. In this study, adult and dominant males used sniffing to track the drops of female urine spread on the substrate bark. During overmarking sessions, the males targeted the drops of urine by tooth-marking or chest rubbing, which can be repeated until the impregnated bark is entirely (or partially) covered by the sternal gland. After targeting females’ depositions, males could drop urine, ending with anogenital rubbing (Figure 3). The fact that males carefully targeted the females’ drops of urine and removed the spot of the females’ anogenital marking suggests the importance of these scent sources in providing honest information [49]. It also indicates the role of overmarking in mate guarding by concealing females’ marks against potential same-sex competitors [55]. This is particularly important if anogenital odours communicate an honest signal about individual absolute and relative major histocompatibility complex (MHC) as found in *Lemur catta* [81]. A previous study comparing genetic heterozygosity to the production of semiochemicals in the scrotal scent gland provided a link between genetics and scent-marking behaviour as a potential advertisement of male quality, suggesting that scent-marking in lemurs provides conspecifics with honest information of their genetic traits [82].

## 5. Conclusions

In summary, the behavioural pattern of scent-marking suggests a strategy to broadcast a mixture of olfactory signals which are deposited at similar heights across individuals and mainly targeting the primarily used substrate part (tree trunk) to effectively provide honest information about depositors’ identity to conspecific group members and individuals from neighbouring groups [55]. The scent mark is deposited in social and sex-specific patterns, with adult and dominant males exhibiting complex scent-marking bouts probably due to the complexity of the signal functions.

Our results suggest that diademed sifakas employ sex-dimorphic mixtures of glandular secretions and excreta to increase the probability of signal transmission and detection by conspecifics. Furthermore, by providing insights into the role of scent sources in honest signalling [49], our study improves the understanding of the vast behavioural repertoire of scent-marking in a wild lemur population.

## Figures and Tables

**Table 1:** Hierarchy dominance of scent depositors and composition of the five study groups in the Maromizaha New Protected Area for the study period. Individuals’ Normalized David’s Score (NormDS) values classify individuals as dominant (DOM) and subordinates (SUB). We removed from the analysis (NA) younger individuals (denoted by a single asterisk) that never participated in negative interactions. We reported females’ and males’ dominance status. F: female, M: male. “+” denotes females that gave birth at least once during the study period. [+18] and [+19] denote individuals that joined the group in 2018 and 2019 respectively, and [−18] and [−19] designate individuals that left the group in 2018 and 2019. AD: adult, SA: sub-adult, Y: young.

Group	ID	Age	Sex	Norm DS	Rank	Slope	Intercept
2PD-MZ	Tandra +	AD	F	3.800	DOM	0.820	4.460
	Onja +	AD	F	2.800	SUB		
	Kintana	AD	F	1.800	SUB		
	Voa	AD	F	1.000	SUB		
	Green *	SA	F	0.600	SUB		
	Kaly [−18]	AD	M	1.667	DOM	0.500	2.000
	Gigi [+18]	AD	M	1.667	DOM	0.667	2.333
	Joby	AD	M	1.000	SUB		
	Tsikivy	AD	M	0.333	SUB		
	Blue *	SA	M	NA	NA	NA	NA
3PD-MZ	Orkide +	AD	F	1.666	DOM	0.500	2.000
	Siramamy +	AD	F	0.667	SUB		
	Rahona [−19]	AD	F	0.667	SUB		
	Andry [+19]	AD	M	1.6667	DOM	0.500	2.000
	Fotsiloha [−19]	AD	M	1.6667	DOM		
	Mike	AD	M	0.6667	SUB		
	Bisky	AD	M	0.6667	SUB		
	Pink *	SA	M	NA	NA	NA	NA
4PD-MZ	Volana +	AD	F	3.800	DOM	0.820	4.460
	Feno	AD	M	2.800	DOM		
	Tantely	AD	M	1.600	SUB		
	Faly	AD	M	1.400	SUB		
	Faniry	SA	M	0.400	SUB		
6PD-MZ	Masina +	AD	F	1.000	DOM	1.000	2.000
	Diane +	AD	F	0.000	SUB		
	Gavo *	Y	F	NA	NA	NA	NA
	Hery	AD	M	1.000	DOM	1	2
	Hasina	SA	M	0.000	SUB		
	Akondro	Y	M	NA	NA	NA	NA
8PD-MZ	Afaka +	AD	F	1.000	DOM	1.000	2.00
	Litchi	SA	F	0.000	SUB		
	Tafita	AD	M	1.667	DOM	0.667	2.333
	Lova	AD	M	1.000	SUB		
	Tia	AD	M	0.333	SUB		
	Papay *	Y	M	NA	NA	NA	NA

**Table 2:** Operational definition of the behavioural component of the scent-marking events recorded across five neighbouring groups of wild diademed sifaka (*Propithecus diadema*) at the Maromizaha New Protected Area, Eastern Madagascar (April–November 2018, May–December 2019). We reported all scent-marking acts including olfactory (denoted by an asterisk) and non-olfactory elements (without asterisk) contributing to the sequence of scent-marking.

Abbreviation	Descriptive Definition of Each Behaviour
SNIFF	When an individual is targeting a substrate, which conspecifics may have scent-marked before, with its nose.
TM	Tooth marking, a non-feeding tree gouging, consists of removing a portion of bark, using the tooth comb. TM is a non-olfactory element related to scent-marking events.
CM *	Chest marking/rubbing consists of pressing the male sternal gland (the chest) and rubbing it against the substrate to deploy the sternal scent odour. This behaviour may occur over substrates and/or females’ urinary and anogenital depositions.
GM *	Anogenital marking/rubbing is observed when an individual presses the anogenital part against the substrate and repeatedly rubs it forward and backwards, to deploy the anogenital gland secretions.
MU *	Urine marking consists of depositing drops of urine (rarely in a stream) over a porous trunk area.
MF *	Faeces marking, where depositors intentionally adhere a few soft faecal pellets on the substrate; anogenital rubbing always follows faecal marking.

**Table 3:** Influence of fixed factors on scent-marking rate: results of a reduced model including only significant interactions. SG-U: scent gland secretion deposited with urine.

Models		Estimate	SE	Z-Value	*p*-Value
GLMM1	(Intercept)	−4.309	0.117	−36.770	<0.001
	Rank: Subordinate	−1.146	0.183	−6.260	<0.001
	Bout: Singular	−0.871	0.149	−5.830	<0.001
	Rank × Bout: Subordinate × Singular	0.755	0.241	3.130	0.002
GLMM2	(Intercept)	−5.175	0.161	−32.110	<0.001
	Rank: Subordinate	−0.432	0.247	−1.750	0.080
	Bout: Singular	−0.067	0.243	−0.280	0.782
	Rank × Bout: Subordinate × Singular	0.165	0.380	0.440	0.663
GLMM3	(Intercept)	−5.917	0.221	−26.804	<0.001
	Rank: Subordinate	−0.246	0.261	−0.941	0.347
	Bout: SG-U	1.021	0.138	7.425	<0.001
	Rank × Bout: Subordinate × SG-U	−0.508	0.218	−2.338	0.019
GLMM4	(Intercept)	−6.095	0.201	−30.326	<0.001
	Rank: Subordinate	−0.042	0.269	−0.157	0.875
	Bout: SG-U	1.298	0.196	6.637	<0.001
	Rank × Bout: Subordinate × SG-U	−0.758	0.279	−2.716	0.007

**Table 4:** Influence of fixed factors on scent-marking occurrence frequency of *Propithecus diadema:* results of a reduced model including only significant interactions.

Models		Estimate	SE	Z-Value	*p*-Value
GLMM5	(Intercept)	−1.3206	0.0876	−15.070	<0.001
	Sequence: GM	0.6010	0.1155	5.202	<0.001
	Sequence: MF	−1.5513	0.1751	−8.860	<0.001
	Sequence: MU	−0.1208	0.1254	−0.963	0.336
	Sequence: TM	−0.1164	0.1253	−0.929	0.353
GLMM6	(Intercept)	0.2682	0.0966	2.778	0.005
	Sequence: MF	−2.8207	0.2012	−14.018	<0.001
	Sequence: MU	−0.9108	0.1394	−6.533	<0.001
GLMM7	(Intercept)	−1.0433	0.0944	−11.049	<0.001
	Sequence: GM	0.1973	0.13067	1.510	0.131
	Rank: Subordinate	−0.5976	0.1454	−4.109	<0.001
	Sequence × Rank: GM × Subordinate	0.8217	0.1909	4.304	<0.001
GLMM8	(Intercept)	0.3196	0.0983	3.251	0.001
	Rank: Subordinate	−0.0854	0.1327	−0.643	0.520
	Sequence: MU	−0.8383	0.1314	−6.379	<0.001
	Sequence × Rank: MU × Subordinate	−0.1581	0.1792	−0.882	0.377

## Data Availability

The data analysed during this study are available from the first author and on GitHub.

## References

[1] Seyfarth R.M., Cheney D.L., Bergman T., Fischer J., Zuberbühler K., Hammerschmidt K. (2010). The Central Importance of Information in Studies of Animal Communication. Anim. Behav..

[2] Fitch W.T., Hauser M.D., Simmons A.M., Fay R.R., Popper A.N. (2003). Unpacking “Honesty”: Vertebrate Vocal Production and the Evolution of Acoustic Signals. Acoustic Communication.

[3] Napier J.R., Napier P.H. (1985). Yawning Serves as a Visual Signal in Primates. The Natural History of The Primates.

[4] Osorio D., Vorobyev M. (2008). A Review of the Evolution of Animal Colour Vision and Visual Communication Signals. Vis. Res..

[5] Campbell-Palmer R., Rosell F. (2011). The Importance of Chemical Communication Studies to Mammalian Conservation Biology: A Review. Biol. Conserv..

[6] Wells D.L., Jensen P. (2017). Behaviour of Dogs. The Ethology of Domestic Animals: An Introductory Text.

[7] Scordato E.S., Drea C.M. (2007). Scents and Sensibility: Information Content of Olfactory Signals in the Ringtailed Lemur, *Lemur catta*. Anim. Behav..

[8] Kleiman D. (1966). Kleiman: Scent Marking in the Canidae. Symp. Zool. Soc. Lond..

[9] Epple G. (1978). Studies on the Nature of Chemical Signals in Scent Marks and Urine of *Saguinus fuscicollis* (Callitricidae, Primates). J. Chem. Ecol..

[10] Epple G., Müller-Schwarze D., Silverstein R.M. (1980). Relationships between Aggression, Scent Marking and Gonadal State in a Primate, the Tamarin *Saguinus fuscicollis*. Chemical Signals: Vertebrates and Aquatic Invertebrates.

[11] Schilling A., Doyle G.A., Martin R.D. (1979). Olfactory Communication in Prosimians. The Study of Prosimian Behavior.

[12] Brown R.E., Macdonald D.W. (1985). Social Odours in Mammals.

[13] Rosell F., Jojola S.M., Ingdal K., Lassen B.A., Swenson J.E., Arnemo J.M., Zedrosser A. (2011). Brown Bears Possess Anal Sacs and Secretions May Code for Sex. J. Zool..

[14] Lewis R.J., van Schaik C.P. (2007). Bimorphism in Male Verreaux’s Sifaka in the Kirindy Forest of Madagascar. Int. J. Primatol..

[15] Caspers J., Radespiel U., Zimmermann E., Schulz S. (2020). Volatile Urinary Signals of Two Nocturnal Primates, *Microcebus Murinus* and *M. lehilahytsara*. Front. Ecol. Evol..

[16] Drea C.M. (2015). D’scent of Man: A Comparative Survey of Primate Chemosignaling in Relation to Sex. Horm. Behav..

[17] Bullard S.C. (1984). Effects of Testosterone upon the Chest-Rubbing Behavior of *Galago Crassicaudatus Umbrosus*. Folia Primatol. Int. J. Primatol..

[18] Lewis R.J. (2006). Scent Marking in Sifaka: No One Function Explains It All. Am. J. Primatol..

[19] Sergiel A., Naves J., Kujawski P., Maślak R., Serwa E., Ramos D., Fernández-Gil A., Revilla E., Zwijacz-Kozica T., Zięba F. (2017). Histological, Chemical and Behavioural Evidence of Pedal Communication in Brown Bears. Sci. Rep..

[20] Jolly A. (1966). Lemur Behavior: A Madagascar Field Study.

[21] Giotto N., Gérard J.-F. (2009). The Social and Spatial Organisation of the Beira Antelope: A Relic from the Past?. Eur. J. Wildl. Res..

[22] Wronski T., Plath M. (2010). Characterization of the Spatial Distribution of Latrines in Reintroduced Mountain Gazelles: Do Latrines Demarcate Female Group Home Ranges?. J. Zool..

[23] Wronski T., Apio A., Plath M., Ziege M. (2013). Sex Difference in the Communicatory Significance of Localized Defecation Sites in Arabian Gazelles (*Gazella arabica*). J. Ethol..

[24] Henry J.D. (1977). The Use of Urine Marking in the Scavenging Behavior of the Red Fox (*Vulpes vulpes*). Behaviour.

[25] Jordan N.R., Golabek K.A., Apps P.J., Gilfillan G.D., McNutt J.W. (2013). Scent-Mark Identification and Scent-Marking Behaviour in African Wild Dogs (*Lycaon pictus*). Ethology.

[26] Mertl-Millhollen A.S. (1979). Olfactory Demarcation of Territorial Boundaries by a Primate—*Propithecus Verreauxi*. Folia Primatol..

[27] Petter J.J. (1962). Recherches Sur l’écologie et l’ethologie Des Lémuriens Malgaches. Mém. Mus. Natl. Hist. Nat..

[28] Boulet M., Charpentier M.J., Drea C.M. (2009). Decoding an Olfactory Mechanism of Kin Recognition and Inbreeding Avoidance in a Primate. BMC Evol. Biol..

[29] Charpentier M.J.E., Williams C.V., Drea C.M. (2008). Inbreeding Depression in Ring-Tailed Lemurs (*Lemur catta*): Genetic Diversity Predicts Parasitism, Immunocompetence, and Survivorship. Conserv. Genet..

[30] Zala S.M., Potts W.K., Penn D.J. (2004). Scent-Marking Displays Provide Honest Signals of Health and Infection. Behav. Ecol..

[31] Palagi E., Dapporto L., Tarli S.B. (2005). The Neglected Scent: On the Marking Function of Urine in *Lemur Catta*. Behav. Ecol. Sociobiol..

[32] Jordan N.R. (2007). Scent-Marking Investment Is Determined by Sex and Breeding Status in Meerkats. Anim. Behav..

[33] Roper T.J., Shepherdson D.J., Davies J.M. (1986). Scent Marking with Faeces and Anal Secretion in the European Badger (*Meles meles*): Seasonal and Spatial Characteristics of Latrine Use in Relation to Territoriality. Behaviour.

[34] Bartecki U., Heymann E.W. (1990). Field Observations on Scent-Marking Behaviour in Saddle-Back Tamarins, *Saguinus fuscicollis* (Callitrichidae, Primates). J. Zool..

[35] Lazaro-Perea C., Snowdon C.T., de Fátima Arruda M. (1999). Scent-Marking Behavior in Wild Groups of Common Marmosets (*Callithrix jacchus*). Behav. Ecol. Sociobiol..

[36] Rosell F. (2002). Do Eurasian Beavers Smear Their Pelage with Castoreum and Anal Gland Secretion. J. Chem. Ecol..

[37] Hayes R.A., Morelli T.L., Wright P.C. (2006). Volatile Components of Lemur Scent Secretions Vary throughout the Year. Am. J. Primatol..

[38] Clapham M., Nevin O.T., Ramsey A.D., Rosell F. (2014). Scent-Marking Investment and Motor Patterns Are Affected by the Age and Sex of Wild Brown Bears. Anim. Behav..

[39] Mills M.G.L., Gorman M.L., Mills M.E.J. (1980). The Scent Marking Behaviour of the Brown Hyaena *Hyaena brunnea*. S. Afr. J. Zool..

[40] Scordato E.S., Dubay G., Drea C.M. (2007). Chemical Composition of Scent Marks in the Ringtailed Lemur (*Lemur catta*): Glandular Differences, Seasonal Variation, and Individual Signatures. Chem. Senses.

[41] DelBarco-Trillo J., Sacha C.R., Dubay G.R., Drea C.M. (2012). *Eulemur*, Me Lemur: The Evolution of Scent-Signal Complexity in a Primate Clade. Philos. Trans. R. Soc. B Biol. Sci..

[42] Ferkin M.H., Johnston R.E. (1995). Effects of Pregnancy, Lactation and Postpartum Oestrus on Odour Signals and the Attraction to Odours in Female Meadow Voles, *Microtus pennsylvanicus*. Anim. Behav..

[43] Greene L.K., Grogan K.E., Smyth K.N., Adams C.A., Klager S.A., Drea C.M. (2016). Mix It and Fix It: Functions of Composite Olfactory Signals in Ring-Tailed Lemurs. R. Soc. Open Sci..

[44] Drea C., Scordato E. (2008). Olfactory Communication in the Ringtailed Lemur (*Lemur catta*): Form and Function of Multimodal Signals. Chemical Signals in Vertebrates 11.

[45] Johnstone R.A. (1996). Multiple Displays in Animal Communication: ‘Backup Signals’ and ‘Multiple Messages’. Phil. Trans. R. Soc. Lond. B.

[46] Palagi E., Norscia I. (2009). Multimodal Signaling in Wild *Lemur catta:* Economic Design and Territorial Function of Urine Marking. Am. J. Phys. Anthropol..

[47] Colquhoun I.C. (2011). A Review and Interspecific Comparison of Nocturnal and Cathemeral Strepsirhine Primate Olfactory Behavioural Ecology. Int. J. Zool..

[48] Boulet M., Crawford J.C., Charpentier M.J.E., Drea C.M. (2010). Honest Olfactory Ornamentation in a Female-Dominant Primate. J. Evol. Biol..

[49] Delbarco-Trillo J., Burkert B.A., Goodwin T.E., Drea C.M. (2011). Night and Day: The Comparative Study of Strepsirrhine Primates Reveals Socioecological and Phylogenetic Patterns in Olfactory Signals. J. Evol. Biol..

[50] Hayes R.A., Morelli T.L., Wright P.C. (2004). Anogenital Gland Secretions of *Lemur catta* and *Propithecus verreauxi coquereli*: A Preliminary Chemical Examination. Am. J. Primatol..

[51] Pochron S.T., Morelli T.L., Terranova P., Scirbona J., Cohen J., Kunapareddy G., Rakotonirina G., Ratsimbazafy R., Rakotosoa R., Wright P.C. (2005). Patterns of Male Scent-Marking in *Propithecus edwardsi* of Ranomafana National Park, Madagascar. Am. J. Primatol..

[52] Dall’Olio S., Norscia I., Antonacci D., Palagi E. (2012). Sexual Signalling in *Propithecus verreauxi*: Male “Chest Badge” and Female Mate Choice. PLoS ONE.

[53] Greene L.K., Bornbusch S., McKenney E.A., Harris R., Gorvetzian S.R., Yoder A.D., Drea C.M. (2019). The importance of scale in comparative microbiome research: New insights from the gut and glands of captive and wild lemurs. Am. J. Primatol..

[54] Norscia I., Antonacci D., Palagi E. (2009). Mating First, Mating More: Biological Market Fluctuation in a Wild Prosimian. PLoS ONE.

[55] Miaretsoa L., Cascella A., Vadàla L., Valente D., De Gregorio C., Torti V., Norscia I., Ratsimbazafy J., Friard O., Giacoma C. (2022). Marking Versus Overmarking: Spatial and Behavioral Patterns of Scent Marking in Wild Diademed Sifaka (*Propithecus diadema*). Int. J. Primatol..

[56] Rasolonjatovo S.M., Irwin M.T. (2019). Exploring Social Dominance in Wild Diademed Sifakas (*Propithecus diadema*): Females Are Dominant, but It Is Subtle and the Benefits Are Not Clear. Folia Primatol..

[57] Irwin M.I. (2020). Propithecus diadema.

[58] Valente D., Miaretsoa L., Anania A., Costa F., Mascaro A., Raimondi T., De Gregorio C., Torti V., Friard O., Ratsimbazafy J. (2022). Comparative Analysis of the Vocal Repertoires of the Indri (*Indri indri*) and the Diademed Sifaka (*Propithecus diadema*). Int. J. Primatol..

[59] Powzyk J.A. (1997). The Socio-Ecology of Two Sympatric Indriids: Propithecus Diadema Diadema and Indri Indri, a Comparison of Feeding Strategies and Their Possible Repercussions on Species-Specific Behaviors.

[60] Randrianarison R.M.S., Rajaonson A., Ralison J.M., Rabemananjara Z., Andrianantenaina T.D., Rabearison J., Ratsimbazafy J. (2015). Local Socio-Economic Effects of Protected Area Conservation: The Case of Maromizaha Forest, Madagascar. Madag. Conserv. Dev..

[61] Altmann J. (1974). Observational Study of Behavior: Sampling Methods. Behaviour.

[62] Shizuka D., McDonald D.B. (2012). A Social Network Perspective on Measurements of Dominance Hierarchies. Anim. Behav..

[63] Patel E. (2012). Acoustic and Olfactory Communication in Eastern Sifakas (Propithecus sp.) and Rhesus Macaques (Macaca mulatta).

[64] R Core Team (2018). R: A Language and Environment for Statistical Computing.

[65] Friard O., Gamba M. Behatrix: Behavioral Sequences Analysis with Permutations Test. http://www.boris.unito.it/pages/behatrix.

[66] Brooks M.E., Kristensen K., van Benthem K.J., Magnusson A., Berg C.W., Nielsen A., Skaug H.J., Mächler M., Bolker B.M. (2017). GlmmTMB Balances Speed and Flexibility among Packages for Zero-Inflated Generalized Linear Mixed Modeling. R J..

[67] Hartig F. DHARMa: Residual Diagnostics for Hierarchical (Multi-Level/Mixed) Regression Models. http://florianhartig.github.io/DHARMa/.

[68] Lüdecke D., Ben-Shachar M.S., Patil I., Waggoner P., Makowski D. (2021). Performance: An R Package for Assessment, Comparison and Testing of Statistical Models. J. Open Source Softw..

[69] James G., Witten D., Hastie T., Tibshirani R. (2013). An Introduction to Statistical Learning: With Applications in R.

[70] Chatterjee S., Simonoff J.S. (2012). Handbook of Regression Analysis.

[71] Forstmeier W., Schielzeth H. (2011). Cryptic Multiple Hypotheses Testing in Linear Models: Overestimated Effect Sizes and the Winner’s Curse. Behav. Ecol. Sociobiol..

[72] Dobson A.J., Barnett A.G. (2002). An Introduction to Generalized Linear Models.

[73] Barr D.J., Levy R., Scheepers C., Tily H.J. (2013). Random Effects Structure for Confirmatory Hypothesis Testing: Keep It Maximal. J. Mem. Lang..

[74] Hothorn T., Bretz F., Westfall P. (2008). Simultaneous Inference in General Parametric Models. Biom. J..

[75] Wyatt T.D. (2010). Pheromones and Signature Mixtures: Defining Species-Wide Signals and Variable Cues for Identity in Both Invertebrates and Vertebrates. J. Comp. Physiol. A Neuroethol. Sens. Neural. Behav. Physiol..

[76] Pochron S.T., Morelli T.L., Scirbona J., Wright P.C. (2005). Sex Differences in Scent Marking in *Propithecus edwardsi* of Ranomafana National Park, Madagascar. Am. J. Primatol..

[77] Kappeler P.M. (1998). To Whom It May Concern: The Transmission and Function of Chemical Signals in *Lemur catta*. Behav. Ecol. Sociobiol..

[78] Hurst J.L., Beynon R.J. (2008). Chemical Signals in Vertebrates.

[79] Lai S.C., Vasilieva N.Y., Johnston R.E. (1996). Odors Providing Sexual Information in Djungarian Hamsters: Evidence for an across-Odor Code. Horm. Behav..

[80] Kappeler P.M. (1990). Social Status and Scent-Marking Behaviour in *Lemur catta*. Anim. Behav..

[81] Grogan K.E., Harris R.L., Boulet M., Drea C.M. (2019). Genetic Variation at MHC Class II Loci Influences Both Olfactory Signals and Scent Discrimination in Ring-Tailed Lemurs. BMC Evol. Biol..

[82] Kappeler P.M., Schäffler L. (2008). The lemur syndrome unresolved: Extreme male reproductive skew in sifakas (*Propithecus verreauxi*), a sexually monomorphic primate with female dominance. Behav. Ecol. Sociobiol..

